# Mucous Secretion and Cilia Beating Defend Developing Coral Larvae from Suspended Sediments

**DOI:** 10.1371/journal.pone.0162743

**Published:** 2016-09-28

**Authors:** Gerard F. Ricardo, Ross J. Jones, Peta L. Clode, Andrew P. Negri

**Affiliations:** 1 Centre for Microscopy, Characterisation and Analysis, The University of Western Australia, Perth, Western Australia, Australia; 2 Australian Institute of Marine Science, Townsville, Queensland and Perth, Western Australia, Australia; 3 Oceans Institute, The University of Western Australia, Perth, Western Australia, Australia; 4 Western Australian Marine Science Institution, Perth, Western Australia, Australia; Department of Agriculture and Water Resources, AUSTRALIA

## Abstract

Suspended sediments produced from dredging activities, or added to the sediment budget via river runoff, are a concern for marine resource managers. Understanding the impact of suspended sediments on critical life history stages of keystone species like corals is fundamental to effective management of coastlines and reefs. Coral embryos (*Acropora tenuis* and *A*. *millepora*) and larvae (*A*. *tenuis*, *A*. *millepora* and *Pocillopora acuta*) were subjected to a range of suspended sediment concentrations of different sediment types (siliciclastic and carbonate) to assess concentration-response relationships on ecologically relevant endpoints, including survivorship and ability to metamorphose. Embryos were subjected to short (12 h) suspended sediment exposures from ages of 3–12 hours old or a long (30 h) exposure at 6 hours old. Neither the survivorship nor metamorphosis function of embryos were significantly affected by realistic sediment exposures to ~1000 mg L^-1^. However, some embryos exhibited a previously undescribed response to dynamically suspended sediments, which saw 10% of the embryos form negatively buoyant cocoons at siliciclastic suspended sediment concentrations ≥35 mg L^-1^. Scanning electron and optical microscopy confirmed the presence of a coating on these embryos, possibly mucus with incorporated sediment particles. Cocoon formation was common in embryos but not in larvae, and occurred more often after exposure to siliciclastic rather than carbonate sediments. Once transferred into sediment-free seawater, functional ~36-h-old embryos began emerging from the cocoons, coinciding with cilia development. Ciliated (> 36-h-old) larvae exposed to suspended sediments for 60 h were also observed to secrete mucus and were similarly unaffected by suspended sediment concentrations to ~800 mg L^-1^. This study provides evidence that mucous secretion and cilia beating effectively protect coral embryos and larvae from suspended sediment and that these mechanisms may enhance their chances of successful recruitment.

## Introduction

Coral reefs provide a range of benefits to coastal communities through tourism, fishing and coastal protection, and have been collectively valued at US $9.9 trillion/yr. globally [1]. However, coral reefs are considered to be in decline due to the impacts of both global (e.g. climate change) and regional (e.g. declining water quality) disturbances [2]. Successful coral reproduction underpins the maintenance of communities and their resilience to disturbance [3, 4]. Of ongoing concern is the increased supply of terrestrial sediment near coral reefs [5], the release of sediments into the water column from dredging activities [6], and the resuspension of sediments from natural wind and wave events [7, 8], and how these stressors may impact coral reproduction and recruitment processes [9–11].

Sediments resuspended from dredging operations can remain elevated for several kilometers, occasionally reaching hundreds of mg L^-1^ (but often <10 mg L^-1^) [12–14]. Similarly, inshore reefs are frequently exposed to suspended sediment concentrations (SSCs) < 5 mg L^-1^, but subject to spikes of >100 mg L^-1^ usually associated with cyclonic activity [15, 16]. These particles have the potential to affect both existing populations of key reef-building taxa, as well as reproduction processes and recruitment of new individuals to these populations[17, 18]. Particularly relevant to managing the risk of dredging projects around coral reefs is the potential for sediment plumes to interact with coral spawning slicks, produced from synchronous, multi-specific release of gametes by broadcasting spawning coral species. For example, since the early 1990s, dredging projects in Western Australia have been required to shut-down all turbidity generating activities (i.e. dredging and disposal of dredge material at sea) shortly before and after synchronous spawning periods [17]. Similar shutdown periods have been implemented on the Great Barrier Reef [19], and have recently been suggested for coral reefs in Singapore [20]. The shutdown policy was introduced under a precautionary principle, which still remains in place, as there are many possible cause-effect pathways whereby suspended sediments (SS) can interact with the reproductive cycle of corals, and few of these have ever been quantified [17]. In particular, the impact of sediment on the planktonic stage remains poorly explored compared with the fertilisation [9–11, 20, 21] and settlement stages, both which often show susceptibility to low sediment levels [22, 23].

**Fig 1:**
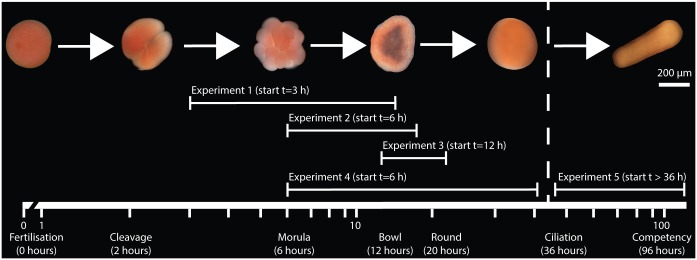
Diagram illustrating the sequence of experiments throughout the embryonic and larval developmental stages of the genus *Acropora*. The experimental design included three experiments (experiment 1, experiment 2 and experiment 3) where embryos were subjected to three separate 12-hour sediment exposures commencing at 3, 6, and 12 hours after fertilisation. In addition, two long sediment exposure experiments (experiment 4 and experiment 5) were conducted covering most of embryogenesis (6–36-h-old embryos) and a 60-hour period following ciliation using larvae at 3–6 days old.

For broadcast spawning corals, the planktonic stage begins following fertilisation of coral gametes at the water’s surface [17]. The first visible signs of embryogenesis generally occur a few hours after fertilisation with the zygote undergoing holoblastic cleavage until four blastomeres are formed [24] (Fig 1). During these initial stages of cleavage, the embryo is increasingly vulnerable to physical disturbance, including fragmentation in turbulent conditions; however, embryonic cells can continue re-dividing resulting in functional, albeit smaller, embryos [25]. Further division of the embryo results in the morula stage, followed in many coral species by a flattened, concave bilayer dish (prawn-chip stage) ~7–9 h after insemination, and then a blastopore formation (bowl stage, Fig 1) [26, 27]. The term ‘embryo’ is used here to denote these developmental stages from fertilisation until blastopore closure. At this stage ciliation and movement occurs [24, 28] and the term ‘larvae’ is used to denote the motile planktonic stage [29]. Like most benthic marine organisms, corals undergo a planktonic larval phase following fertilisation generally lasting 4–10 days (reviewed by Jones et al. [17]), although larger larvae and those that acquire algal symbionts, *Symbiodinium* spp., have the greatest potential to disperse long distances through energy derived from lipids or supplied via photosynthesis [30, 31]. The larval stage ends when the larvae permanently attach to a substratum and undergo metamorphosis [29]. At a behavioural level, coral exhibit a sensory capacity to identify sites that are suitable for settlement, but are limited in their ability to navigate towards reefs and therefore are considered mostly planktonic [32–35].

A range of responses of planktonic stages to SS have been reported. Humphrey et al. [21] found no difference in developmental abnormalities in very early stage embryos (~four-cell stage) of 3-h-old *Acropora millepora* after sediment exposures of 200 mg L^-1^. Similarly, Gilmour [10] found no clear trends in survivorship of 3–18 h-old embryos of *A*. *digitifera* exposed to ~100 mg L^-1^. At the larval stage, Te [36] did not observe any larval mortality of the brooding coral *Pocillopora damicornis* subjected to 1000 mg L^-1^ SSC, but Gilmour [10] found significant mortality of *A*. *digitata* larvae at much lower SSC of ~50 mg L^-1^. More recently, in a pilot study by Larsson et al. [37] observed a decrease in larval survivorship of *Lophelia pertusa* at ~ 25 mg L^-1^ SSC.

Some of the variability in the outcome of these studies could be methodological, with some approaches unlikely to achieve a uniform, consistent suspension of sediments throughout the course of the experiment [17]. Gilmour [10] used coarse silt to fine-grained sediments, whereas Larsson et al. [37] removed coarse-grain sediment through a two day settlement process to select only for very fine-grained particles for testing. The broadcast spawning *Lophelia pertusa* larvae are much smaller (~20%) in length than the brooding *P*. *damicornis* larvae, and therefore likely to contain comparatively less energy reserves that may be drawn upon to overcome sediment encounters. *L*. *pertusa* larvae used by Larsson et al. [37] is also a cold water species, as opposed to the tropical species used by Gilmour [10], Humphrey et al. [21], and Te [36].

As part of an experimental sequence to investigate the effects of sediments on the early life-history stages of corals and understand the risk associated with turbidity-generating events during coral spawning periods [9, 11, 17], we examined the survivorship and metamorphosis response of embryo and larvae of several tropical coral species after exposure to different sediment types and concentrations. The quantitative approach used in this study, and derivation of concentration–response relationships, will allow more informed assessment of the risk, as opposed to hazard (sensu Harris et al. [38]), of SS on the planktonic phase of corals.

## Materials and Methods

### Sediment collection and preparation

Experiments were conducted with two types of marine sediments: predominantly siliciclastic sediments collected from Onslow Reef (Pilbara region, Western Australia: 21°38’32 S, 114°55’27 E), and predominantly carbonate sediments collected from Davies Reef, (Great Barrier Reef, Queensland: 18°49’12 S, 147°39’21 E). Collection and preparation of the field-collected sediment have been described in Ricardo et al. [9]. Briefly, the sediments were screened, milled and settled until the modal grain size was <10 μm as measured using laser diffraction techniques (Mastersizer 2000, Malvern instruments Ltd). Each type of sediment had relatively low proportions of total organic carbon (0.26%). Suspended sediment treatments were created by making a serial dilution of the concentrated stock with 0.4 μm filtered seawater (FSW), and the resultant turbidity (NTU) measured with a nephelometer (TPS 90FL-T) and spectrophotometer (Shimadzu, UV-1800). Sediment concentrations in the samples were determined by spectrophotometry at the start and end of the experiments, or when a water change was conducted (typically every 12 h). For each treatment, 3.5 mL of the sample was measured for absorbance at 820 nm and the mean of the initial and final absorbance readings calculated. Turbidity and absorbance values both correlated linearly with SSCs (R² > 0.98) and were therefore used to derive total SSCs in the chambers. To confirm SSCs for each experiment, 3 × 100 mL replicate samples of the highest concentration were filtered through 0.4 μm polycarbonate filters (Advantec), which were dried overnight in an oven at 60°C and the sediments weighed on an analytical balance to 0.0001 g. During the experiments, salinity (35.5 ppt) and pH (8.1) remained constant. Dissolved oxygen was measured in the highest sediment treatment and remained above 95% saturation. All experiments were carried out in a temperature controlled room set at the same temperature as the outdoor aquaria (27–29°C depending on the month of spawning), and the water temperature within the chambers did not deviate from this range.

### Coral collection and larval culture

Colonies of *Acropora millepora* (Ehrenberg, 1834), *A*. *tenuis* (Dana, 1846), and *Pocillopora acuta* (Lamarck, 1816) were collected from <10 m depth 3–5 days before the predicted spawning events from 2013–2015 in the central Great Barrier Reef from inshore and mid-shelf reefs (19°10’13 S, 146°51’53 E; 18°22’53 S, 146°47’43 E; 18°49’12 S, 147°39’21 E; 18°48’48 S, 147°39’26 E; 18°46’25 S, 146°31’07 E) (S1 Table). All corals were collected under the Great Barrier Reef Marine Park Authority Permit G12/35236.1.

Gravid colonies were transported to the National Sea Simulator at the Australian Institute of Marine Science (AIMS), and placed in outdoor flow-through seawater tanks of 27–29°C (equivalent to the water temperature at their collection site). At the ‘setting stage’ just prior to spawning (see Babcock and Heyward [39]), colonies were isolated in individual tanks and egg-sperm bundles gently scooped from the water surface after spawning. The embryo and larval culture procedures were conducted following methods described in Negri and Heyward [40]. Briefly, gametes were cross-fertilised for ~1.5 h in 20 L of 0.4 μm FSW in a 50 L container. The embryos were then washed free of sperm by gently transferring them into another 50 L container also containing FSW. This process was repeated three times. Embryos were then transferred into 500 L fiberglass tanks filled with FSW, where they were left to develop for 12 h, after which time gentle aeration and water flow was introduced to provide adequate water circulation and maintain sufficient dissolved oxygen levels.

For the brooder *P*. *acuta*, larval traps were placed on tanks containing adult colonies on the night of the new moon in April 2014. Over the following seven mornings, larvae were collected from the traps and transferred to 5 L glass chambers containing FSW, with gentle aeration and water flow.

### Embryo concentration–response experiments

Embryos of *A*. *millepora* were exposed to a range of SSCs (up to ~1,000 mg L^-1^) for 12 h, in 3 separate experiments, starting with embryos 3 h after fertilisation (3–15 h, experiment 1), 6 h after (6–18 h, experiment 2) and 12 h after (12–24 h, experiment 3) (Fig 1). A longer-term (30-h) experiment was also conducted at a range of lower SSCs (<100 mg L^-1^) starting with embryos 6 h after fertilisation (6–36 h, experiment 4). The control chambers contained no sediment but all other conditions were identical to the treatment chambers. We assessed embryo survivorship in all experiments, and in experiment 2 and 3 we assessed numbers of embryos forming cocoons, and in experiment 4 we assessed the ability of larvae to settle following the exposure of embryos to sediments. The SSC ranges were selected to span the maximum running-mean values embryos could encounter for a given exposure duration based on the analyses of water quality conditions during three major capital dredging projects [13] and for peaks in instantaneous turbidity measurements during one capital dredging project (see below). Specifically, we covered the maximum SSCs for the relevant durations of exposure. For example, shorter exposure durations (i.e. 12 h) result in higher 100% running means (i.e. hundreds of mg L^-1^), whereas longer exposures (1–3 days) result in lower 100% running means (tens of mg L^-1^). Between 10–20 embryos were added to chambers containing 150 mL of each SS treatment and placed on mechanical rollers at 0.3 revolutions s^-1^ to maintain the sediments in suspension. Every few hours the chambers were gently inverted a few times and then placed back on the mechanical rollers. After exposure, the total number of surviving embryos was counted, with damaged, missing, and inert embryos defined as dead. To test the ability of larvae to undergo normal metamorphosis, embryos previously subjected to SS were transferred into FSW for a further 5-day recovery period, and after this time the larvae were considered competent to settle (competency generally commences after 4 days [17]). These 6-day-old larvae were then exposed to a 2 × 2 mm chip of live crustose coralline algae (CCA, *Hydrolithon onkodes*) to assess their ability to settle and undergo metamorphosis [29]. The experiment was repeated using *A*. *tenuis* embryos of the same age to examine replicability between species.

### Larval survivorship and metamorphosis concentration–response experiments

Larval survivorship was examined in >3-day old *A*. *millepora*, *A*. *tenuis* and *P*. *acuta* larvae subjected to sediment suspensions to ~800 mg L^-1^ over a period of 60 h (Fig 1). As with the embryo assays, sediment concentrations used in the larval experiments spanned the range of environmentally relevant concentrations expected for 1–3 day exposures. The control chambers contained no sediment but all other conditions were identical to the treatment chambers. For each sediment concentration, 20 larvae were added to each of 4 × 180 mL chambers containing 150 mL of the sediment suspension. Sediments were kept in suspension by rotating the chambers at 0.3 revolutions s^-1^ using mechanical rollers, and the resuspension was assisted by the use of three 6 × 6 × 75 mm rods attached to the inner-surface of the chambers (as baffles) to disturb the water movement. Every few hours the chambers were gently inverted a few times and then placed back on the mechanical rollers. Every 12 h, the sediment suspensions were changed, and the larvae assessed for survivorship at the end of the experiment. Surviving larvae were then gently washed and transferred to 6-well tissue culture plates containing 10 mL of FSW, and at 6-day old were assessed for their ability to undergo attachment and metamorphosis using CCA chips, as described previously (except for *P*. *acuta* where metamorphosis rates were poor in the controls—see ‘test acceptability criteria’ below).

### Optical and scanning electron microscopy

Embryos and larvae at the end of the experiments were examined using light microscopy, and by scanning electron microscopy (SEM) using samples fixed in 1.25% glutaraldehyde and 0.5% paraformaldehyde in FSW. The SEM samples were subsequently dehydrated in a microwave using a graded ethanol series for 40 s at 250 W and then critical point dried (Polaron KE3000, Quorum Technologies) in liquid CO_2_. The dried samples were mounted on carbon tape on aluminium stubs, coated with 3 nm platinum, and imaged using a field emission SEM (Zeiss 55-VP). Backscattered signals are proportional to atomic composition and therefore these images were used to identify sediments on the larval samples. Elements of greater atomic number, such as calcium, iron, and silicon appeared bright relative to the sample [41] and the biological material are primarily composed of elements with low atomic numbers (carbon, oxygen and hydrogen), and appeared dark in the sample.

### Water quality during turbidity-generating events

A year of water quality turbidity readings (1/6/2010 to 31/5/2011) were examined to determine the ephemeral nature of SS pulses and brief reprieves from SS exposure. Turbidity readings were collected with sideways mounted optical backscatter devices (nephelometers) at two water quality coral reef sites Site 1 (LNGA: 20° 49.322’ S, 115° 30.665' E) and Site 2 (LNGO: 20° 49.713’ S, 115° 30.507’ E) subjected to periodic cyclone events and ~300 m from a major capital dredging program at Barrow Island, Western Australia. For further water quality collection and sites details see Ricardo et al. [9], and Jones et al. [13].

### Statistical Analysis

We defined the test acceptability criteria as experiments that had a high rate of survivorship (>80%) and metamorphosis (>50%) in the controls, and only these were included for analysis [42]. Suspended sediment concentrations that resulted in a 10% (EC_10_) or greater response in absolute terms were calculated where possible, by fitting the data to non-linear regression curves (four-parameter logistic models) with 95% confidence bounds using the software GraphPad Prism (v7), and by fitting binomial Generalized Linear Models (GLM) using a probit link using the statistics program R (v3.1.2). Non-linear regression (NLR) models were only fitted under the criteria that they passed normality of residuals and the replicates test (a measure of deviation from the model) [43]. GLM models were corrected for overdispersion using quasi-likelihood estimations [44] and EC_X_ values extracted using the package dose.p [45]. For easier interpretation, GLM models were only plotted if NLR models could not be fitted. Four chambers leaked during experiment 5 leading to a loss of larvae and therefore these data were not included in the analyses. All sample size analyses were conducted *a priori* for binomial GLM using G*Power (v3.1.9.2). Pilot experiments indicated high survivorship (>90%) in the controls and low cocoon formation (0%), and therefore because the response could only be unidirectional e.g. survivorship cannot be >100% [46]), survivorship and cocoon formation were run as one-tailed hypotheses with α = 0.05, β = 0.8. Post-hoc analysis confirmed our sample sizes were sufficient. For settlement assays, which were considered two-tailed hypotheses, a lower settlement rate in the control than expected in the *A*. *millepora* embryo and *A*. *tenuis* larval experiments meant that our minimum detection effect was 13 and 16% respectively at α = 0.05, β = 0.8.

In situ 10-min turbidity data were converted to approximate SSCs using the conversion factor of 1.3 NTU: SSC [9]. The data were analysed using Matlab (v8.6) for sediment pulse durations above 35 mg L^-1^ (a SSC required for the formation of embryo cocooning to occur—see Results).

## Results

### Impacts of suspended sediments on embryogenesis

**Fig 2:**
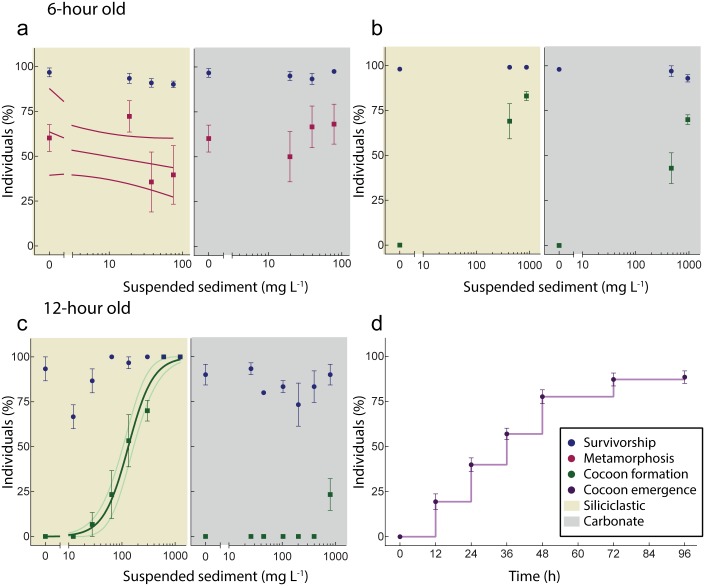
Concentration-response relationships for *A*. *millepora* embryos. a) Survivorship and ability to metamorphose after prolonged exposure to siliciclastic (yellow shade) and carbonate (grey shade) suspended sediment (SS) from 6–36 h age, n = 6 per concentration. b) Survivorship and cocoon formation after exposure to a 12-h sediment exposure of siliciclastic (yellow shade) and carbonate (grey shade) SS from 6–18 h age. c) Survivorship and cocoon formation after exposure to a 12-h sediment exposure of siliciclastic (yellow shade) and carbonate (grey shade) SS from 12–24 h age. d) Larval emergence from the cocoon after exposure to 12-h sediment exposure (siliciclastic sediment only, n = 12 per time interval). Data points staggered for visualization. Each replicate contains 10–20 embryos.

The 3-h old embryos exposed for 12-h to ~800 mg L^-1^ SSC (experiment 1) fragmented upon agitation (used to keep the sediment suspended) and so it was not possible to quantify survivorship. There was no effect of exposure of 6-h-old embryos to either siliciclastic or carbonate sediments at concentrations up to ~80 mg L^-1^ for 30 h (experiment 4, Fig 2a). The SS exposure did not have any significant effect (GLM: b = -0.1761, t = -1.209, p = 0.204) on the subsequent ability of the larvae to metamorphose following a 4.5-day recovery period, despite a 29% decrease in settlement rates (S2 Table, Fig 2a). The experiment was repeated using 6-h-old embryos subjected to SSCs of ~900 mg L^-1^ for 12 h, and there was also no effect on survivorship in either sediment type (experiment 2, Fig 2b). Exposure of 12-h-old embryos for 12 h to elevated SSCs (siliciclastic: ~1200 mg L^-1^; carbonate: ~800 mg L^-1^) had no effect on survivorship for either sediment type (experiment 3, Fig 2c), although in the 10 mg L^-1^ exposures, embryos sometimes clumped with mucus, causing a decrease in survivorship in some chambers (Fig 2c). The high SSC exposures of ~1000 mg L^-1^ had no impact on subsequent metamorphosis (S1 Fig). In *A*. *tenuis*, a single exposure experiment on 12-h-old embryos at siliciclastic SSCs to ~1000 mg L^-1^ had no impact on survivorship (S1 Fig).

**Fig 3:**
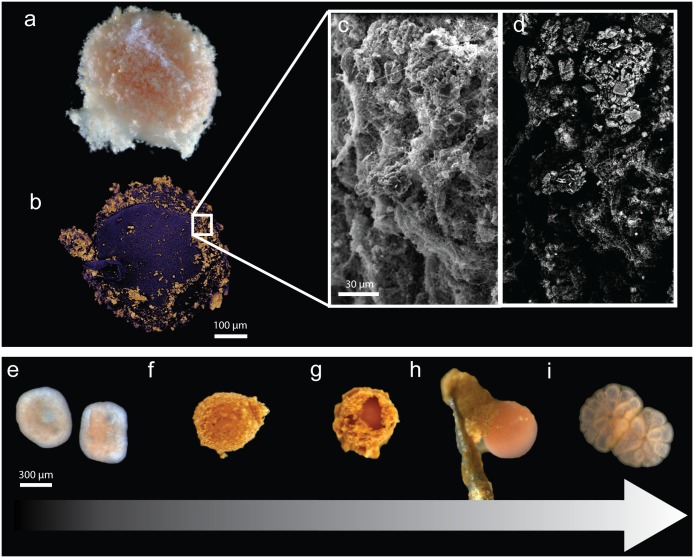
Microscopy of *A*. *millepora* embryos in mucous cocoons. a) a mucous cocoon under optical microscopy following exposure to carbonate sediment, b) a false-colored backscatter electron image of a scrapped mucous cocoon showing sediment (yellow) bound the embryo (purple), c) secondary electron image showing the mucous coating (high contrast) and d) backscatter electron image showing sediment grains (high contrast). Progression of mucous cocoons through development, e) early developmental stages (i.e. bowl stage at 12 h old) embryos before sediment exposure, f) mucous cocoons during sediment exposure (the orange color of the cocoon reflects the orange color of the siliciclastic sediment used), g) ciliated larva spinning and tearing open the cocoon, h) larva emerging from the cocoon (with assistance using a dissection probe for photograph), i) larvae (6 days old) were capable of metamorphosis once competent.

**Fig 4:**
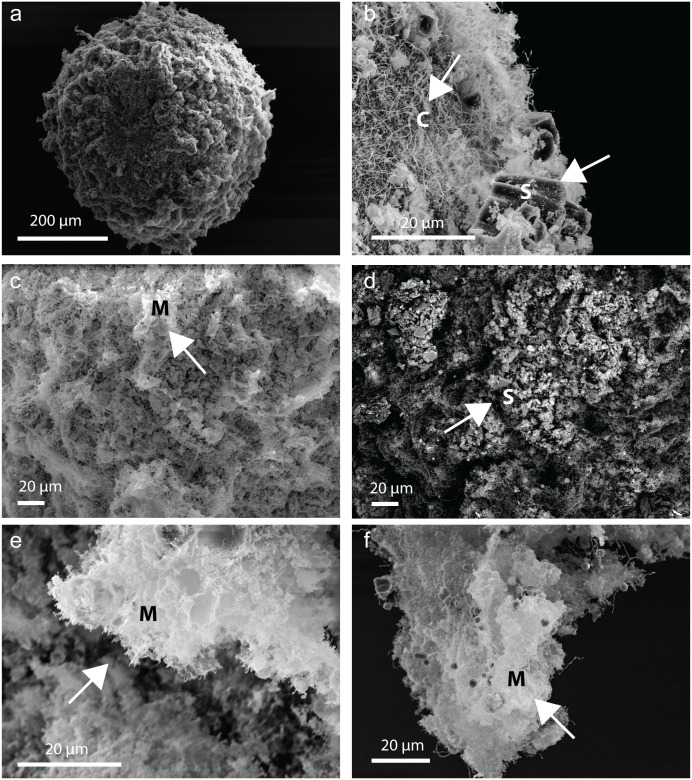
Scanning electron microscopy images of the mucous cocoon around an embryo of *Acropora millepora*. a) Image of the cocoon showing a thick mesh enveloping the embryo. b) With part of the cocoon removed, cilia can be observed developing underneath. A closer inspection of the cocoon showing c) a stringy web interpreted as mucus and d) bound sediment grains clearly revealed under backscatter electron microscopy. e-f) Thick protrusions of mucus could be seen throughout parts of the cocoon.

Although there was no effect of SS on survivorship, embryos often formed ‘cocoons’ that quickly became negatively buoyant and resulted in the embryos sinking (Fig 3a). The color of the cocoon reflected the colour of the sediment grains (Fig 3a and 3f), and under SEM the embryo cocoon appeared to be a casing composed of sediment grains incorporated in mucus (Figs 3b–3d and 4a–4e). Once transferred to FSW, the larvae were able to free themselves from the cocoon, with the movement generated by newly developed cilia (Fig 3g and 3h and S1 File). For 6-h-old embryos, cocooning was observed in high proportions at the high SSC (Fig 2b). Cocoon formation was observed in 12-h-old embryos exposed to the siliciclastic sediments with 10% (EC_10_) of the embryos forming cocoons at 35 mg L^-1^ (95% C.I.; 20–60) (S2 Table, Fig 2c), but embryos of the same age showed less sensitivity to carbonate sediment with mucous cocooning (23 ± 7%, mean ± SEM) only observed in the highest (~800 mg L^-1^) sediment concentration (Fig 2c). In sediment-free FSW, the entrapped larvae were first observed emerging from the cocoon after 12 h and by 48 h, >75% had emerged (Fig 2d). After emerging from the cocoon, the larvae were capable of swimming and undergoing normal metamorphosis (S1 Fig).

Cocoon formation was observed as late as 72 h after fertilisation in the development sequence in *A*. *millepora*, but only in a few individuals (data not shown). Exposure to siliciclastic SS for a brief 1-h period elicited some cocooning in embryos. Cocoon formation was also observed for *A*. *tenuis* embryos subjected to siliciclastic SSC at ~600 mg L^-1^, with all emerging from the cocoon within 4 days (S1 Fig). However, embryos of either species did not create the cocoons without water movement and attempts to recreate mucous cocoons by inverting the chambers every 5 min (a less effective method for sediment resuspension) were largely unsuccessful. As a final examination of the cocoon formation, commercially available high grade processed calcium-bentonite clay (Watheroo Bentonite) was tested and caused all embryos to form cocoons at SSC as low as 20 mg L^-1^ and inverting the chambers every 5 min was capable of inducing cocoon formation at bentonite treatment of ~100 mg L^-1^.

### Impacts of suspended sediments on larval development and metamorphosis

**Fig 5:**
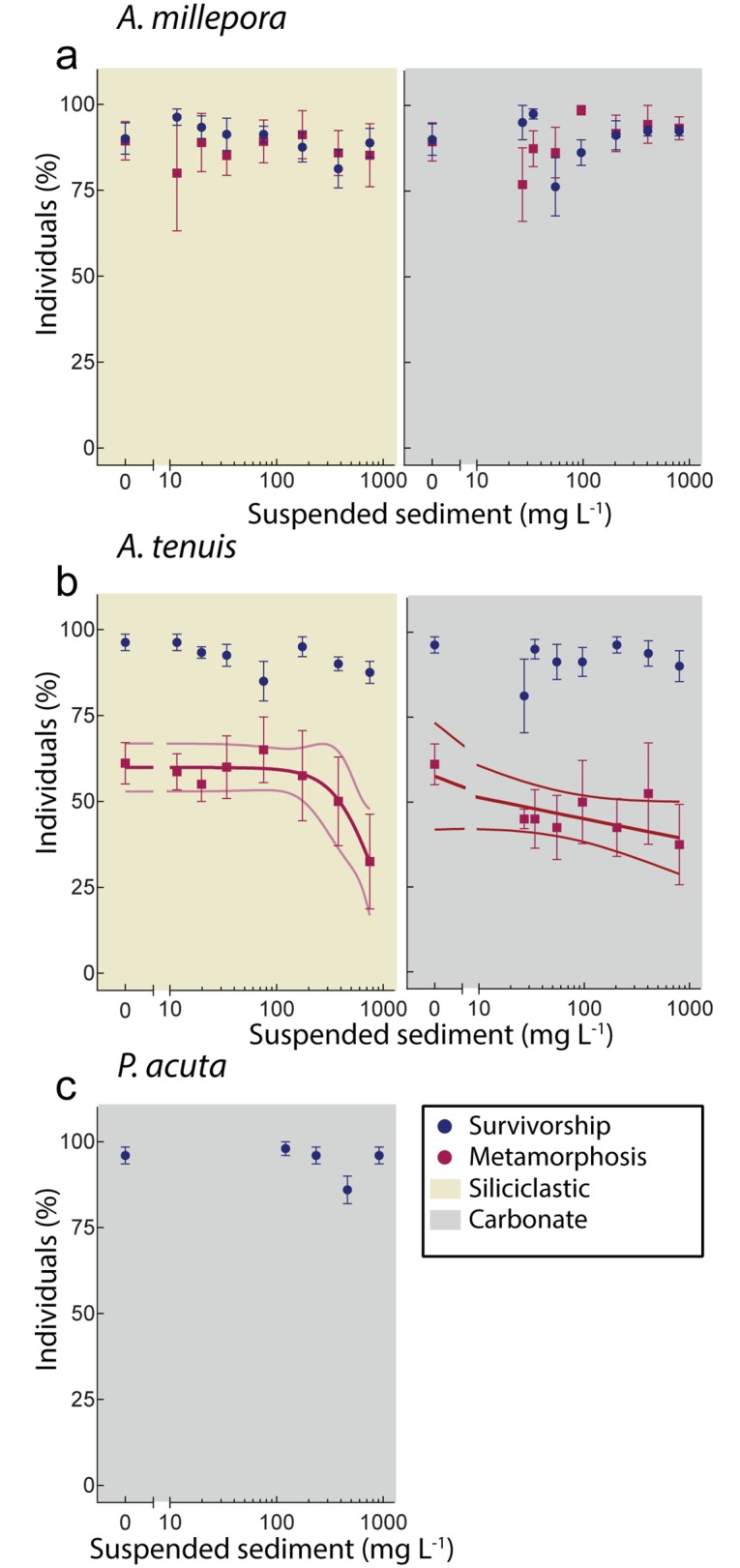
Concentration-response relationships between suspended sediments (SS) and larval survivorship and ability to undergo metamorphosis. a) Survivorship and metamorphosis of > 3-day-old larvae *A*. *millepora* following sediment exposure to siliciclastic (yellow shade) and carbonate (grey shade) sediment. c) Survivorship and metamorphosis of > 3-day-old larvae *A*. *tenuis* following sediment exposure to siliciclastic (yellow shade) and carbonate (grey shade) sediment. e) Survivorship of *P*. *acuta* following sediment exposure to carbonate sediment. Data points staggered for visualization. Each replicate contained 10–20 larvae, with n = 4–5 per concentration.

**Fig 6:**
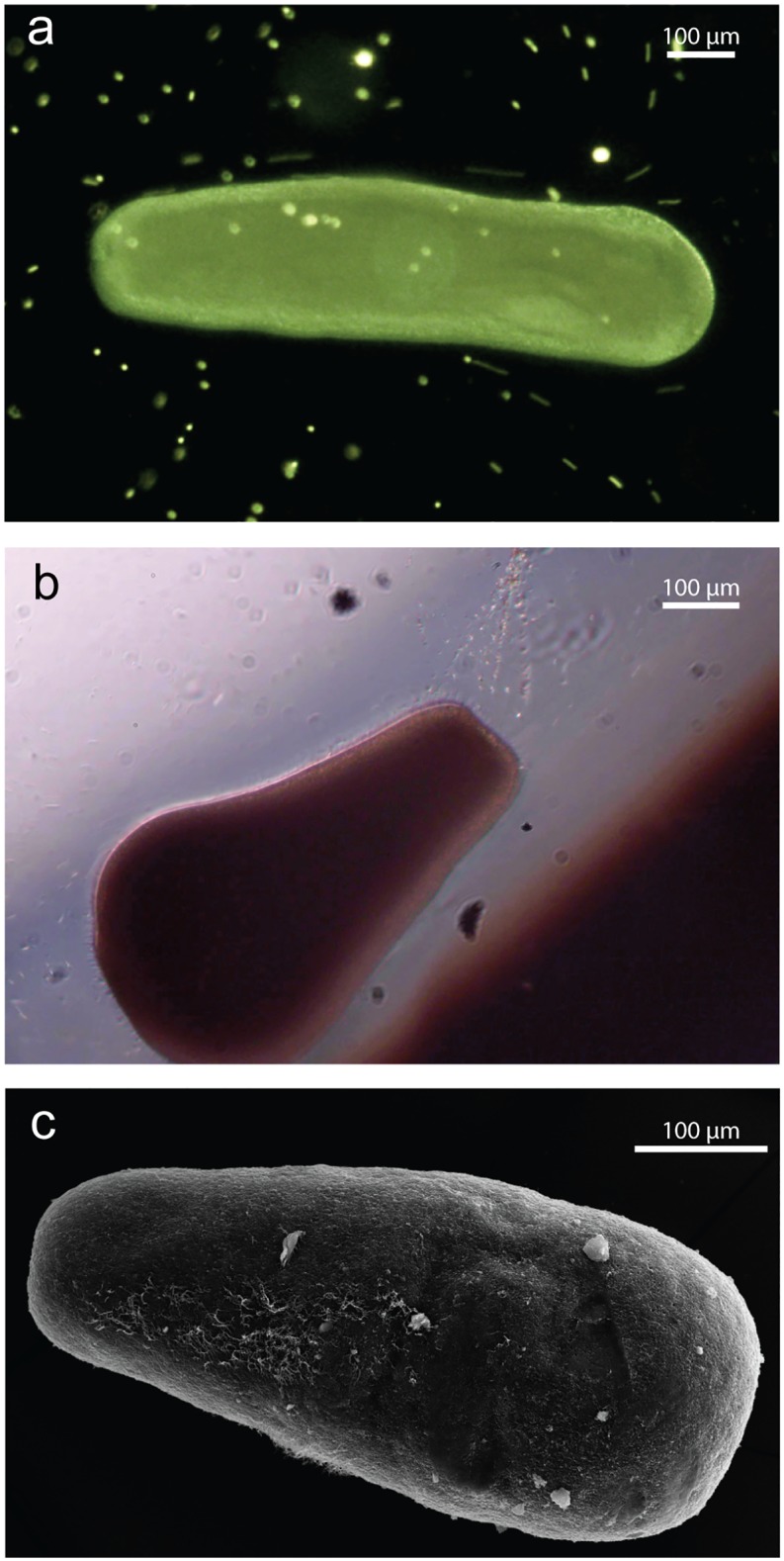
Observations of sediment removal mechanisms of *A*. *millepora* larvae under optical and scanning electron microscopy. a) florescence microscopy of a larva deflecting florescent beads through cilia beating b) optical microscopy of a larva clearing sediment through mucous production, and c) scanning electron microscopy of a larva after being exposed to elevated SS for 60 h showing few grains adhering to its surface.

There was no effect of either sediment type on survivorship or ability to metamorphose of >3-day-old larvae of *A*. *millepora*, even at very elevated SSC (~800 mg L^-1^), and for extended exposure durations (~60 h) (Fig 5a). Similarly, no effect on survivorship was observed for *A*. *tenuis* using either sediment type (Fig 5b). Upon transferring to clean FSW, siliciclastic sediments at high concentrations caused a non-significant decrease in the ability of larvae to metamorphose (GLM: b = -0.182, t = -1.780, p = 0.085), and a similar non-significant trend was observed for larvae exposed to carbonate sediment (GLM: b = -0.1581, t = -1.540, p = 0.134) (S2 Table, Fig 5b). The survivorship of *Pocillopora acuta* larvae was not affected by exposure of carbonate sediments up to ~900 mg L^-1^ (Fig 5c). Optical microscopy revealed larvae actively cleared sediment grains and florescent beads through cilia beating (Fig 6a and 6b) in addition to some mucous secretion (Fig 6b and S2 File) and scanning electron images revealed few sediment grains adhered to larvae (Fig 6c).

### Water quality during turbidity-generating events

Turbidity peaks during dredging and natural events are very episodic, with turbidity remaining above 35 mg L^-1^ (the concentration required to form mucous cocoons) for a mean of 49 min (average of both sites) (S2 Fig). The time at these elevated concentrations was highly skewed to brief peaks lasting <1.5 h (~80%). In total, there were 595 turbidity events above 35 mg L^-1^ at Site 1 and 456 turbidity events at Site 2 over the course of the year.

## Discussion

Coral embryos and larvae are relatively resilient to elevated concentrations of SS, employing impressive protective strategies such as mucous production and cilia beating to assist in the removal or avoidance of sediments. A novel mechanism was observed which involved the encapsulation of embryos in a sediment–mucous layer under dynamic resuspension conditions, with the planula shedding the cocoon once ciliated. There was no obvious legacy of damage impacting further larval development and metamorphosis under realistic sediment exposure concentrations and durations.

Suspended sediments in dredge plumes constitute a hazard to developing embryos and coral larvae, but whether they constitute a risk depends on the SSCs generated, the probability of encountering those conditions and the sensitivity of embryos and larvae to the sediments [17, 38]. Recent analyses of temporal and spatial patterns in water quality during several large-scale capital dredging programs have emphasized the very high variability in SSCs and the transient natures of plumes (S2 Fig) [13, 14]. Close to dredging, concentrations can reach hundreds of mg L^-1^, but these high values are typically short-lived events (i.e. a few hours). Over time periods which are more relevant for the planktonic phase of coral larvae (i.e. days), the upper percentiles of SSCs are typically a few tens of mg L^-1^ [6]. These water quality values were derived from fixed optical backscatter nephelometers, measuring turbidity as a proxy for SSCs in passing plumes. Coral embryos and larvae could encounter and drift with highly turbid plumes and may therefore be subject to high concentrations for longer durations than recorded by fixed devices. Taking a conservative approach, embryos and larvae were exposed to very high SSCs (up to ~800 mg L^-1^) over periods of several days. Even under these high conditions, and in response to two very different sediment types, there was no obvious effect on survivorship of embryos and ciliated larvae of two broadcast spawning *Acropora* spp. and mature planulae of the brooding species *Pocillopora acuta*. Importantly, there was also no subsequent impact of the sediments on the ability of the larvae to metamorphose when transferred to sediment-free clean seawater.

For the brooded larvae, these results support the earlier study of Te [36], who also did not see any effects on survivorship in larvae of *P*. *damicornis* at SSCs of up to 1,000 mg L^-1^. However, the results differ from the study of Gilmour [10], who found effects on survivorship as low as ~50 mg L^-1^ in the broadcast spawning species *Acropora digitifera*. As discussed in Jones et al. [17], there may have been a range of water quality issues associated with the incubation chambers used in the study, in particular the possibility of stagnation and reduced water exchange caused by the suspended sediment and high larval concentrations. Preliminary studies with the *Acropora* species used here, indicated that if water was not exchanged regularly with FSW (i.e. every 12 h), mortality of a few larvae quickly resulted in the loss of all remaining larvae in the chamber (Ricardo personal observation). Larsson et al. [37], who reported effects on larval survivorship of the deep-water coral *Lophelia pertusa* at low concentrations (25 mg L^-1^), also reported issues with their methodology including handling stress and low sample size. In their opportunistic study (from an unexpected spawning event), many larvae disappeared on the first day of the study and their analysis was based on changes in larval survivorship from one day after adding the larvae until the end of the 5-day experiment. The authors emphasized the need to conduct more extensive assessment of the effects of sediment exposure to examine the reproducibility of the pilot study. We suggest that conditions listed above may have led to an overestimation of the sensitivity of the larvae to SS. However, the sediments used in the current study were relatively nutrient-poor, relevant to those found along the Western Australian coastline or beneath the substrate biofilm layer. Sediments occurring in nutrient-rich waters, or in combination with other stressors, may have a greater impact on the pelagic stages. Further, differences between species in terms of energy reserves or ability to remove sediment grains may also lead to species-specific responses.

Our results indicate that coral embryos and larvae may be less sensitive to elevated SSCs than other early life stages and processes. Here, there was no obvious decline in survivorship or larval metamorphosis after sediment exposure within realistic environmental concentrations. We previously demonstrated a potential 10% impact on coral bundle ascent at concentrations as low as 47 mg L^-1^ [11], whereas effects on fertilisation have been reported at SSCs as low as 35–100 mg L^-1^ [10, 11, 21]. Coral embryos and larvae appear well equipped to deal with elevated sediment particles, either repelling them by beating cilia, or removing them by mucous secretion, which represents a novel protection mechanism in coral larvae. Secretion of a mucosubstance was commonly observed throughout embryogenesis and larval stages during exposure to SS. In adult corals, mucous production is associated with feeding processes (i.e. mucous entrapment), and in conjunction with ciliary movement, for self-cleaning and manipulating sediments that have settled on the coral’s surfaces [47, 48]. Both mucous production and ciliary movement have a latent, sub-lethal energetic cost [49] although the cost of cilia beating in corals is believed to be negligible compared with the energetic cost of respiration [50]. Overall the ciliary beating and mucous production did not translate to any obvious impairment of larval development or the ability of the larvae to metamorphose once transferred to sediment-free seawater.

Under elevated SSCs, the early developing embryos became covered in a layer of the mucosubstance that completely cocooned the embryo. The accumulation of sediment grains on the cocoon quickly sunk the embryo, typically within 2 h of exposure. Within the cocoon, the early-stage embryos underwent normal larval development, eventually forming cilia which resulted in their movement and spinning inside the casing. After the larvae were removed from sediment exposure, they were capable of shedding the cocoon and completing development into functional larvae and undergoing metamorphosis. Thus, cocoon formation around embryos appeared to act as a mechanism for protection, and removal of sediment in the absence of cilia. The formation and shedding of the cocoons adds to a number of ways mucus is utilised in marine organisms. Mucous cocooning has previously been observed in some fish of the suborder Labroidei, as a means to protect against parasites and predators [51, 52], and mucous secretion is a common physiological process in adult corals as a response to stress including exposure to sediment [6, 53]. Some adult colonies of the genus *Porites* can occasionally form thick, viscid ‘sheets’ of mucus on their surfaces, which can ultimately envelope the whole colony [54, 55], and capture sediments, algae and debris [56]. The mucous sheet eventually sloughs off the colony’s surface by water movement, thereby removing the sediment [53, 55]. However, the use of mucus during early life history stages is less understood. During broadcast spawning events, coral egg-sperm bundles are wrapped in a mucous membrane that packages the gametes and it is hypothesized the mucus is secreted from the eggs [57]. Previously, we demonstrated that sediment grains can bind to the bundle membrane and coarse grains may sink and delay the egg-sperm bundle from reaching the surface where fertilisation takes place [11]. In some corals, embryogenesis occurs close to the surface of the adult coral, usually trapped within a mucous matrix [24, 58, 59]. The matrix was described as adhesive and adhered to objects it contacted [58]. At the other end of the pelagic life-history stage, mucous secretion is hypothesized to aid in the attachment of the larva to substrata during settlement [53].

In this study, cocooning was commonly observed during the embryo stage but few, if any, were observed once the larvae were ciliated and neither was cocooning observed earlier on eggs exposed for 3-h to SS (S3 Fig). Therefore, it is unlikely the cocoon is a remnant part of the bundle mucous membrane. Recently, a hyaline layer (which assists in cellular orientation of embryos during development) has been proposed for one species of coral *Tubastraea coccinea*, but this has yet been identified in other species [60]. At these very early stages of development, it is unlikely mucous-producing cells have been formed, and in *Acropora millepora* these tend to increase in numbers typically after ~170 h [26]. An attempt to stain the cocoon with Alcian Blue (which stains acidic polysaccharides) was unsuccessful but may have failed because coral mucous composition can vary in carbohydrate, proteins and lipids [53, 61]. Another possibility is that the mucosubstance secretes directly from the ectoderm.

Mucous cocoons formed on 10% of embryos in the presence of siliciclastic SSCs as low as ~35 mg L^-1^. Despite the average sediment concentration near dredging operations remaining elevated over longer periods [13], SS pulses >35 mg L^-1^ were usually short in duration, often lasting < 1h. Sharp decreases in SSCs between sediment pulses may offer larvae in mucous cocoons a brief reprieve to split and emerge from the cocoon. If, however, the sediment concentration remains elevated or sediment deposition rate is high, the embryos or larvae could remain entrapped and smothered. Cocoons created in response to carbonate sediments required greater concentrations of SS, probably owing to these sediments being less abrasive and sticky in nature. In contrast, only 20 mg L^-1^ of highly sticky bentonite clay caused all embryos to form mucous cocoons. With the exception of bentonite clay, embryo mucous cocoons only formed under constant agitation, and especially under unidirectional movement. Therefore, naturally suspended particles in the absence of water movement is unlikely to create mucous cocooning and locations with high agitation such as inshore wave-swept shorelines that have abrasive or sticky components in the sediment may be necessary to activate this response.

The formation of sediment-mucous cocoons has implications for the larval dispersal stage of the coral lifecycle. Most dispersal between reefs is through self-recruitment (philopatric), but larvae are capable of travelling considerable distances (teleplanic), which may increase genetic diversity and assist in the transition of coral populations to higher latitudes as water temperatures increase [30, 62, 63]. Many larvae of broadcast spawning corals become competent and recruit between 4–10 days [17], and the sinking of the embryos in sediment-mucous cocoons in conditions of high turbidity may reduce the pelagic phase by 1–2 days, restricting dispersal, and therefore may limit the ability of distant reefs to recover from disturbances. Further, these larvae may be restricted to settlement in unfavorable areas near their natal reef, which may be subject to ongoing elevated sediment levels. Other cause-effect pathways may affect embryo and larval dispersal phases that were not investigated in the study. Reductions in light associated with SS may confuse phototactic responses of larvae, entrainment of circadian rhythms [64] and the combined impact of downward sediment flux and increased cilia beating (to deflect sediment grains) may interfere with vertical positioning in the water column, ultimately impacting on dispersal and settlement [17]. Moreover, larval exposure to sediment may carry a legacy of impact on life-history stages beyond settlement. However, assessing the consequences of these impacts in both the laboratory and the field remains a challenge.

## Conclusion

Newly developing embryos and ciliated larvae were robust to high SSCs used in this study, with no impact on larval survivorship or their ability to metamorphose observed. Combined, both life-history stages demonstrated an ability to remove sediment grains and tolerate high sediment loads. Therefore, SS related risks to the embryo and larval stages should be considered of lower concern when compared with more sediment-sensitive life-history stages such as fertilisation and settlement [11, 22, 23], which bracket the pelagic stages.

## Supporting Information

S1 Fig Survivorship, cocoon formation and settlement of 12-h-old embryos after 12-h exposure to siliciclastic sediment. Survivorship, cocoon formation and settlement of a) *Acropora millepora* and b) *Acropora tenuis* embryos. Settlement was assessed after the ciliated larvae emerged from the cocoon and had developed until competency. No settlement data were presented for *A*. *tenuis* because of insufficient rates in the control.
(TIF)

S2 Fig The duration of NTU-derived suspended sediment concentrations remaining above 35 mg L^-1^ at two water quality monitoring sites ~300 m from a major capital dredging program (~7.6 M m^3^ of sediment dredged over 530 d at Barrow Island. a) Site 1 was located 300 m north and b) Site 2 located 300 m south. Dredging operations were suspended for 12 days from 20–31 March 2011 for the coral spawning environmental window and for a few days associated with the close proximity of cyclones Bianca, Dianne and Carlos.
(TIF)

S3 Fig Microscopic examination of the *Acropora tenuis* eggs after 3-h sediment exposure. (TIF)

S1 File Larva of *A*. *millepora* spinning in a mucous cocoon shortly before emerging. (MP4)

S2 File Larva of *A*. *millepora* deflecting sediment grains and producing mucus. (MP4)

S3 File Raw data for experiments conducted in the study. (XLSX)

S1 Table Sites and dates of coral collections. (DOCX)

S2 Table Summary table of experiments with a >10% decline in response compared to the control. (DOCX)
