# Supplementary figures and images for: Mucous Secretion and Cilia Beating Defend Developing Coral Larvae from Suspended Sediments
Source: PLoS One. 2016 Sep 28;11(9):e0162743. doi: 10.1371/journal.pone.0162743 (PMC5040398; doi:10.1371/journal.pone.0162743)

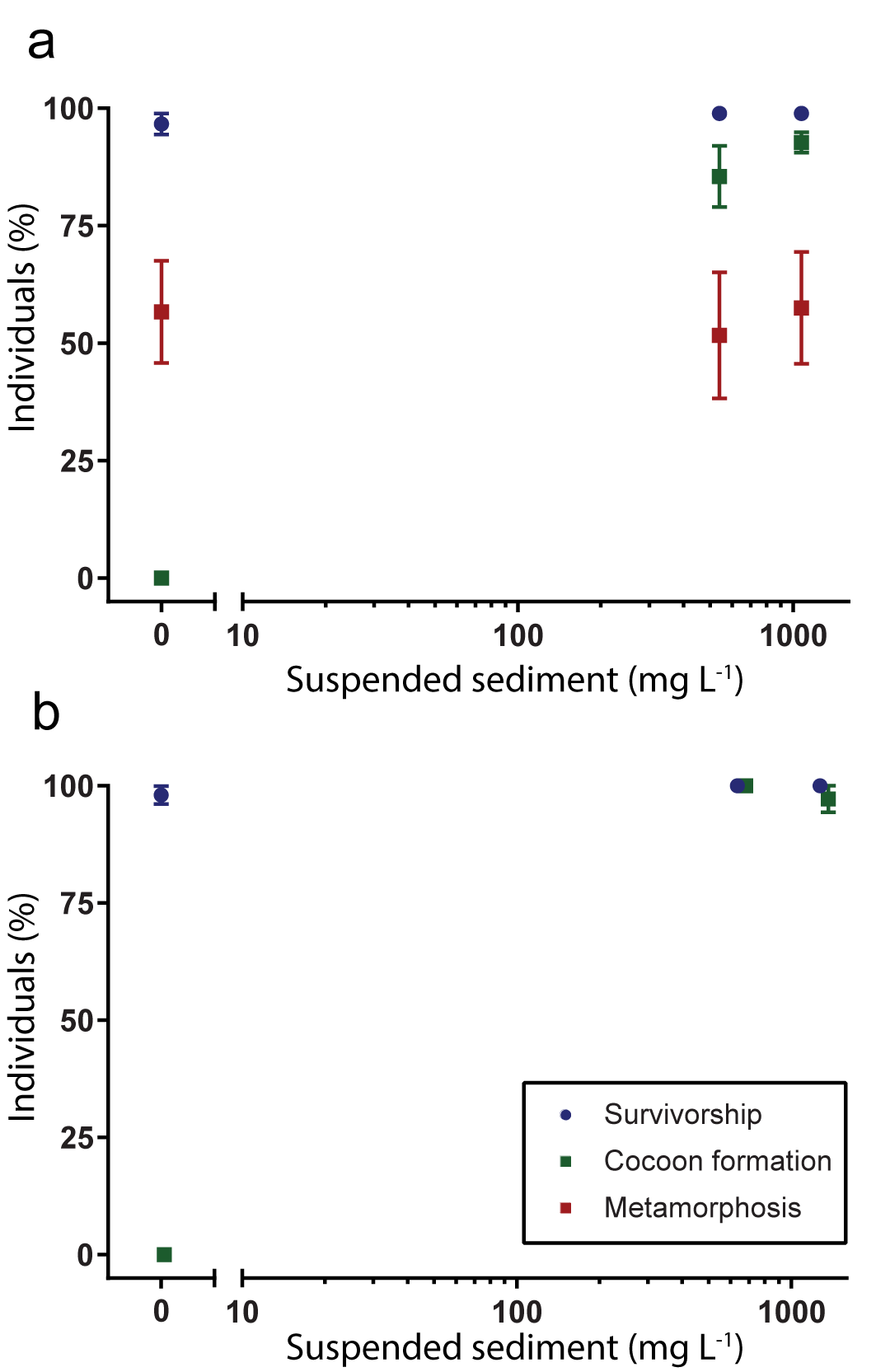

Supplement: S1 Fig — Survivorship, cocoon formation and settlement of a) Acropora millepora and b) Acropora tenuis embryos. Settlement was assessed after the ciliated larvae emerged from the cocoon and had developed until competency. No settlement data were presented for A. tenuis because of insufficient rates in the control. (TIF) [file pone.0162743.s001.tif]

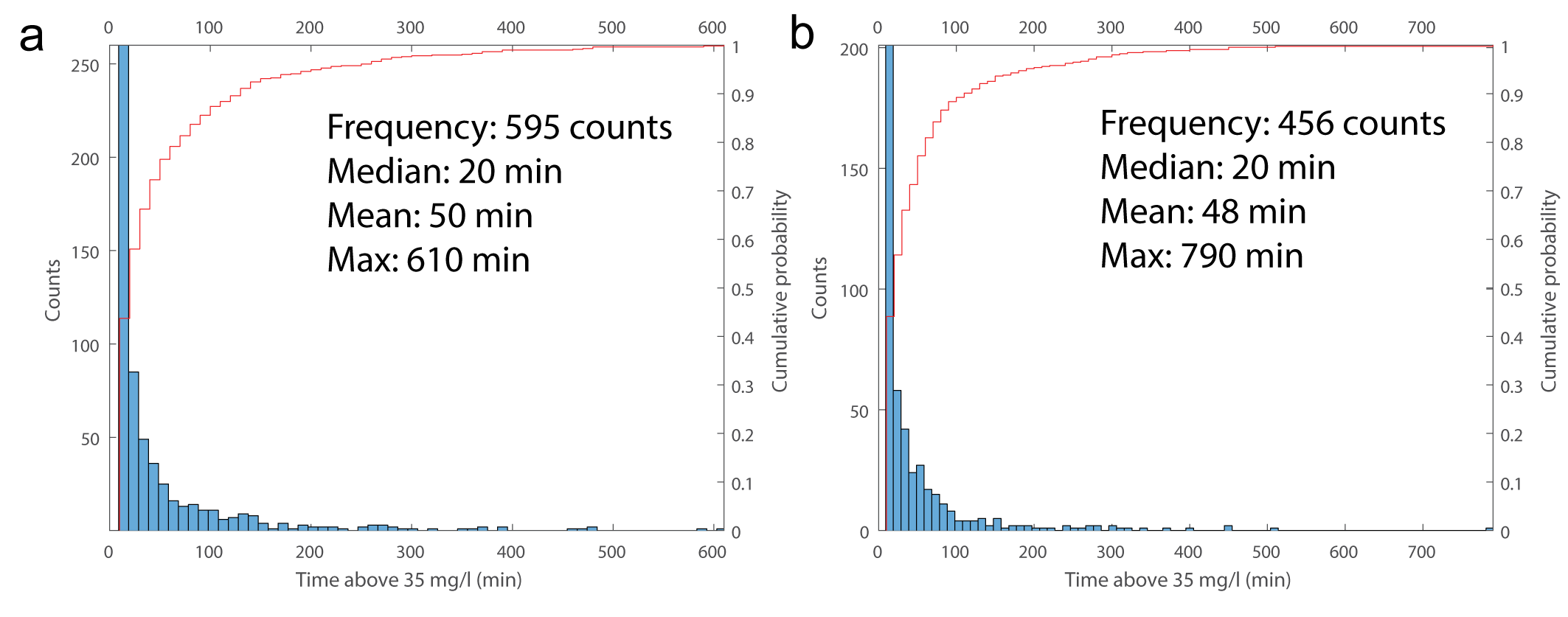

Supplement: S2 Fig — a) Site 1 was located 300 m north and b) Site 2 located 300 m south. Dredging operations were suspended for 12 days from 20–31 March 2011 for the coral spawning environmental window and for a few days associated with the close proximity of cyclones Bianca, Dianne and Carlos. (TIF) [file pone.0162743.s002.tif]

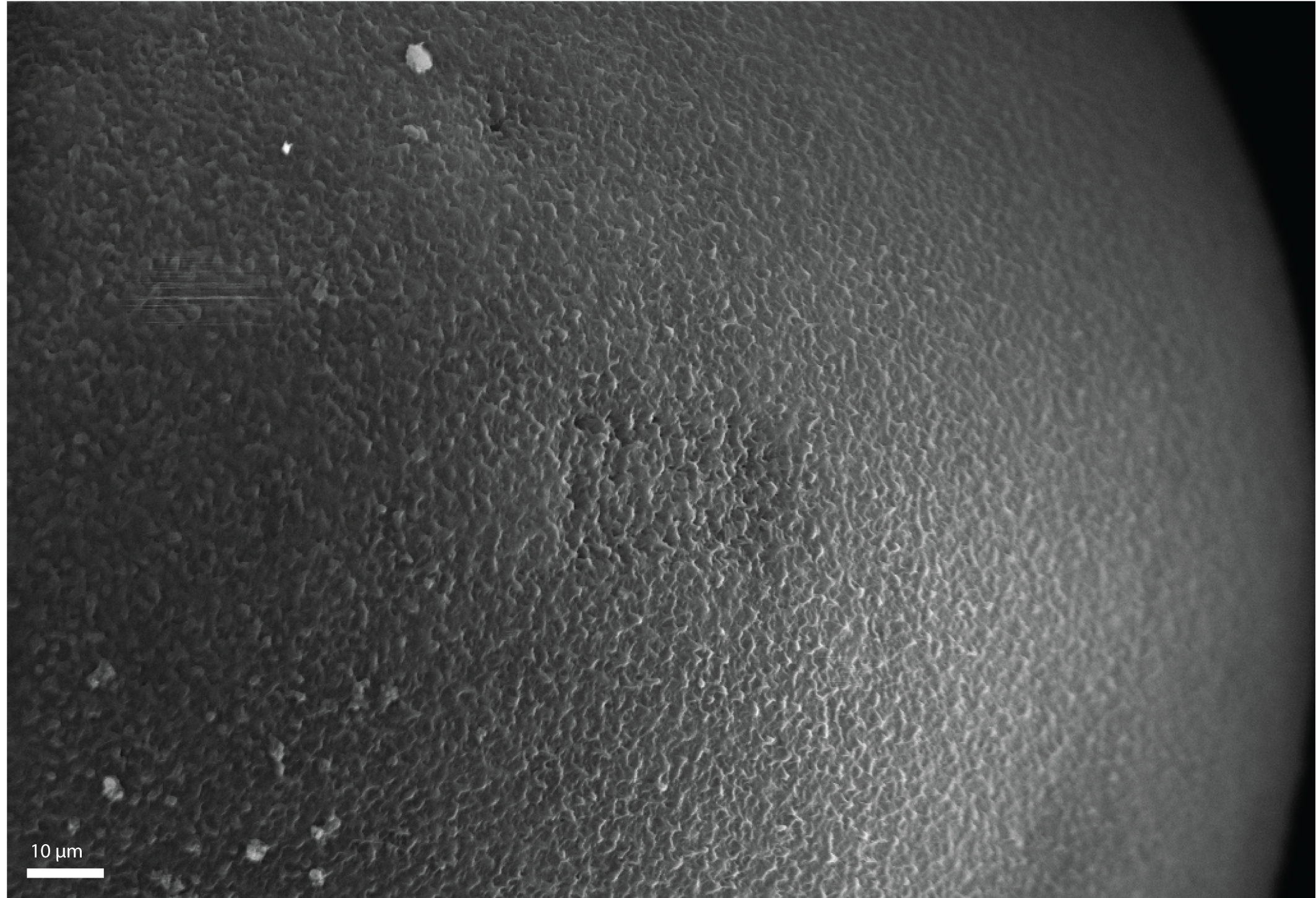

Supplement: S3 Fig — (TIF) [file pone.0162743.s003.tif]
