# Supplementary material for: Mucous Secretion and Cilia Beating Defend Developing Coral Larvae from Suspended Sediments
Source: PLoS One. 2016 Sep 28;11(9):e0162743. doi: 10.1371/journal.pone.0162743 (PMC5040398; doi:10.1371/journal.pone.0162743)
Supplement: S1 Table — (DOCX) [file pone.0162743.s007.docx]

**S1 Table.** **Sites and dates of coral collections.**

| **Collection sites** | **Latitude/Longitude** | **Location** | **Species collected** | **Dates** |
| --- | --- | --- | --- | --- |
| **Magnetic Island** | (19°10’13 S, 146°51’53 E) | Central, inshore | *Acropora tenuis* | 21 October 2013  6 October 2014 |
| **Trunk Reef** | (18°22’53 S, 146°47’43 E) | Central, mid-shelf | *Acropora tenuis*, *Acropora millepora* | 14–17 November 2013, 3 November 2014, 10 December 2014, 19–21 November 2015 |
| **Davies Reef** | (18°49’12 S, 147°39’21 E), (18°48’48 S, 147°39’26 E) | Central, mid-shelf | *Acropora tenuis*, *Acropora millepora*, *Pocillopora acuta* | 12 February 2014 (*P. acuta* only), 10 December 2014, 19–21 November 2015 |
| **Esk Reef** | (18°46’25 S, 146°31’07 E) | Central, mid-shelf | *Acropora tenuis*, *Acropora millepora* | 26–27 October 2015 |
