# Supplementary material for: Mucous Secretion and Cilia Beating Defend Developing Coral Larvae from Suspended Sediments
Source: PLoS One. 2016 Sep 28;11(9):e0162743. doi: 10.1371/journal.pone.0162743 (PMC5040398; doi:10.1371/journal.pone.0162743)
Supplement: S2 Table — (DOCX) [file pone.0162743.s008.docx]

**S2 Table. Summary table of experiments with a >10% decline in response compared to the control.**

| **Experiment** | **EC10 ± 95% CI**  **(mg L^-1^)** | **EC20 ± 95% CI**  **(mg L^-1^)** | **EC50 ± 95% CI (mg L^-1^)** | **P-value** |
| --- | --- | --- | --- | --- |
| **Ability of *A. millepora* to metamorphose after embryos exposed to siliciclastic SS (Fig 2a)** | NLR: N/A | NLR: N/A | NLR: N/A | N/A |
|  | GLM: 0.87 (0.10 – 246)*,** | GLM: 7.0 (0.10 – 476)* | GLM: N/A | 0.204 |
| **Cocoon formation of *A. millepora* after embryos exposed to siliciclastic SS (Fig 2c)** | NLR: 35 (20 –55) | NLR: 57 (38 – 81) | NLR: 134 (104 –173) | N/A |
|  | GLM: 40 (28–57) | GLM: 59 (44 – 80) | GLM: 129 (103 – 162) | <0.001 |
| **Ability of *A. tenuis* to metamorphose after larvae exposed to siliciclastic SS (Fig 4b)** | NLR: 300 (1 – N/A)* | NLR: 431 (1 – N/A) | NLR: N/A | N/A |
|  | GLM: 9.6 (1.0 – 163)*,** | GLM: 83 (9.0 – 764)* | GLM: N/A | 0.085 |
| **Ability of *A. tenuis* to metamorphose after larvae exposed to carbonate SS (Fig 4b)** | NLR: N/A | NLR: N/A | NLR: N/A | N/A |
|  | GLM: 8.3 (0.2 – 298)*,** | GLM: 69 (6 – 793)* | GLM: N/A | 0.134 |

*Denotes when EC_x_ value occurs within 95% CI of the control. ** Denotes an effect size within the minimum detection limit. N/A Denotes when a model could not be fitted, or the EC_x_ value occurs outside of the range of the concentrations tested.
